# A Bayesian Approach to Predict Food Fraud Type and Point of Adulteration

**DOI:** 10.3390/foods11030328

**Published:** 2022-01-25

**Authors:** Jan Mei Soon, Ikarastika Rahayu Abdul Wahab

**Affiliations:** 1 Faculty of Allied-Health and Wellbeing, University of Central Lancashire, Preston PR1 2HE, UK; 2 Faculty of Agro-Based Industry, University Malaysia Kelantan, Jeli 17600, Kelantan, Malaysia; ikarastika@umk.edu.my

**Keywords:** artificial enhancement, Bayesian network, chemicals, inspections, manufacturing, mislabelling, retailer

## Abstract

Primary and secondary food processing had been identified as areas vulnerable to fraud. Besides the food processing area, other stages within the food supply chain are also vulnerable to fraud. This study aims to develop a Bayesian network (BN) model to predict food fraud type and point of adulteration i.e., the occurrence of fraudulent activity. The BN model was developed using GeNie Modeler (BayesFusion, LLC) based on 715 notifications (1979–2018) from Food Adulteration Incidents Registry (FAIR) database. Types of food fraud were linked to six explanatory variables such as food categories, year, adulterants (chemicals, ingredients, non-food, microbiological, physical, and others), reporting country, point of adulteration, and point of detection. The BN model was validated using 80 notifications from 2019 to determine the predictive accuracy of food fraud type and point of adulteration. Mislabelling (20.7%), artificial enhancement (17.2%), and substitution (16.4%) were the most commonly reported types of fraud. Beverages (21.4%), dairy (14.3%), and meat (14.0%) received the highest fraud notifications. Adulterants such as chemicals (21.7%) (e.g., formaldehyde, methanol, bleaching agent) and cheaper, expired or rotten ingredients (13.7%) were often used to adulterate food. Manufacturing (63.9%) was identified as the main point of adulteration followed by the retailer (13.4%) and distribution (9.9%).

## 1. Introduction

The increasing scale, the complexity of food supply networks, and current disruptions due to COVID-19 and climate variability can lead to food and drink products becoming more vulnerable to fraud. Food fraud is the intentional modification of food products for financial gain. Factors that influence food fraud range from resource scarcity to inadequate governance and low probability of detection [1]. Food fraud can occur anywhere in the food supply chain i.e., pre-farm level (e.g., counterfeit seeds), in the raw material, in an ingredient, as well as in the final product or the food packaging, to catering services (e.g., substitution or misrepresentation of dishes). Although it is difficult to quantify the impact on the whole food supply chain, Spielman (2020) [2] estimated the impact of food fraud on the food industry to be in excess of $50 billion annually. Food and drink categories most commonly affected by fraud include dairy products [3,4], meat products [3,5], seafood [3,6], alcohol products [3,6], and fats and oils [6,7]. The type of adulterants used in dairy products includes nitrogen sources (e.g., ammonium salts, melamine, urea, and non-dairy proteins) [8,9] to mask the reduction of dairy protein content caused by dilution. Substances such as formaldehyde, hydrogen peroxide, hypochlorite, and salicylic acid are also added to enhance product shelf-life [8,10].

Counterfeiting applies to intellectual property rights i.e., rights given to the creator for the exclusive use of his/her creation for a time period and are comprised of trademark, patent, copyright, and trade secret [11,12]. Counterfeiting activities in food include the sale and manufacture of products using a trademark without the permission of the brand’s owner [13]. Counterfeiting accounts for more than 40% of identified fraud in the beef supply chain including processing and packing of meat in unapproved premises, products were produced without inspection, and/or documents such as entry and health certificates were either forged or missing [5]. Meat substitution is another form of adulteration in beef products, including substituting with pork, turkey [14], horse [15], and offals [16]. Prediction of food fraud using Bayesian Networks (BN) has been carried out using regional databases such as Rapid Alert System for Food and Feed (RASFF) and Economically Motivated Adulteration incidents (EMA) [17,18]. Yang et al. 2019 [19] identified beverages, alcohols, and processed fruit and vegetable products as risky food in China market using Bayesian modeling and meta-analysis.

BN are probabilistic graphical models that represent a set of variables and their probabilistic dependencies. It uses the relationship between variables to compute probability [20]. Bouzembrak and Marvin, 2016 [17] developed a Bayesian network model that predicted 80% of the fraud types correctly while Marvin et al., 2016 [18] identified fish and seafood, meat and fruits and vegetables as the product categories with the highest probabilities of fraud. The latter study successfully demonstrated the application of BN as a holistic approach as the study included many drivers of fraud (e.g., price of the product, trade volume, country of origin, indices for perceived corruption, price spike during a period of fraud) and data sources from RASFF and EMA. Fruits and vegetables from China were most commonly affected by artificial enhancement such as the use of unauthorized pesticides [21]. Accurate prediction of food fraud, targeted food category, and country of origin is beneficial to border controls and inspections. In addition to these, it will benefit food authorities and the food industry to predict the point of adulteration, i.e., at which stage was the food or drink product adulterated. Robson et al., 2020 [5] reported that primary processing is the most vulnerable area in beef fraud. Processing techniques such as mincing, filleting, grinding, crushing, chopping is required to facilitate the production of a variety of food and drink products. However, it is at this point where the original form of the food products is altered and is indistinguishable from other similar food ingredients. This makes primary and secondary processing extremely vulnerable to fraudulent activities. Besides the processing area, other stages within the food supply chain such as farms [22] and food services [23] are also vulnerable to fraud. This is evident in van Ruth et al., 2020 [23] who reported casual dining food services as being most vulnerable to fraud. Organized crime such as theft in farms is also becoming prevalent [22].

Food fraud seems to be prevalent throughout the whole food supply chain. Besides primary and secondary processing, where are the hotspots in the food supply chain that are vulnerable to fraud? This study defines the point of adulteration as ‘when food or drinks were intentionally modified (e.g., substituted, diluted, artificially enhanced, misrepresented during processing and packaging) or when fraudulent activities took place (e.g., theft during distribution, forged documents at border controls) for financial gain. This research will answer the following questions: Can we predict the types of food fraud and food categories targeted by fraudsters? At what points were food and drinks subjected to fraud? Answering these questions will help to fulfill the knowledge gaps and strengthen the global food supply chains’ resilience against food fraud and during exogenous shock events like COVID-19. This study aims to develop a Bayesian network model to predict food fraud type and point of adulteration. The BN model could potentially benefit food authorities and the food supply chain to prioritize vulnerable areas or hotspots for fraudulent activities and implement food fraud mitigating strategies accordingly.

## 2. Materials and Methods

### 2.1. Food Adulteration Incidents Registry (FAIR) Database

A total of 893 incidents reported in the Food Adulteration Incidents Registry Database were reviewed from 1979–2018. FAIR Database is hosted by the US Food Protection and Defense Institute (FDPI) and collates global data on economically motivated adulteration and incidents of deliberate tampering. Users could access information over five years old for free [1,24]. FAIR Database includes both food fraud and food threat incidents (e.g., food tampering incidents associated with extortions, mental illness, malicious and religiously, ideologically or politically motivated intents). Note that only food fraud data and one tampering record (for economic gain purposes) were used in this study. The main difference between FAIR and RASFF databases was RASFF data were mainly food safety issues although it also includes reports of food adulteration. Most of the incidents reported in the FAIR Database were also captured in Decernis Food Fraud Database (previously USP Food Fraud Database).

Incident number, year reported, food category, food or drink products, incident type, type of adulteration, type of adulterants or hazards, reporting country, point of adulteration, point of detection, and summary of the incidents were recorded. One of the benefits of using the FAIR database was accessed to information on point of adulteration i.e., where potential adulterants were added or point of fraud occurred. Duplicates (*n* = 29) and tampering incidents due to malicious, religious, ideological, or politically motivated events (*n* = 149) were removed from the list. With the exemption of one record, all tampering incidents associated with food threats were removed as the focus of this study was to predict food fraud type due to the intentional modification of food or drink products for financial gain and not malicious intent. Only one tampering incident was included in the BN model. This was a unique event where the perpetrator contaminated a water reservoir with pesticides to increase the sale of his water purifiers [24].

Spink and Moyer 2011 [25] identified seven types of food fraud i.e., adulteration, counterfeit, diversion, over-run, simulation, tampering (for economic gain purpose), and theft. Adulteration is further divided into addition, dilution (for liquid-based products), substitution, and artificial enhancement. According to Li et al., 2020 [26] and Zhang and Xue 2016 [27], artificial enhancement is defined as the addition of unapproved chemical additives and/or addition of substances to artificially enhance the quality or attributes of a product. Manning and Soon 2016 [28] and Soon and Manning 2018 [29] included misleading indications on labels or packaging and smuggling or illegal trafficking of food as fraud. Based on the evaluation of the incident summary in the FAIR database and the methodology described by Marvin et al., 2016 [18], this study identified addition, adulteration, artificial enhancement, counterfeit, dilution, diversion, intentional distribution of unacceptable food, mislabelling, smuggling, substitution, tampering (for economic purposes), theft and transshipment. Adulteration was included when no indication on how the food or drink products were adulterated were reported in the incident summary. Further descriptions of each food fraud category are described in Table 1.

### 2.2. BN Model

**Figure 1:**
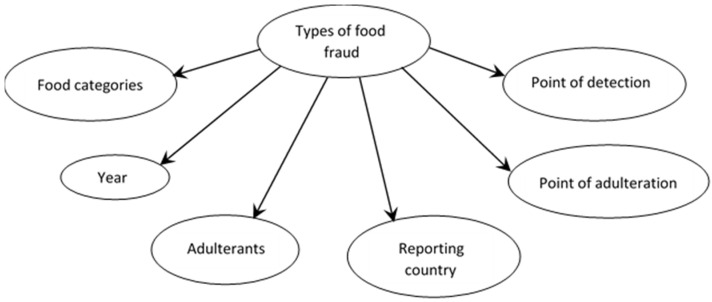
Bayesian Network (BN) model of types of food fraud and explanatory variables.

Types of food fraud were linked to six explanatory variables (Figure 1). Details of the explanatory variables are shown in Table 1. The probability of food fraud event $F_{i}$ at the condition of event $F_{j}$, $P\left(F_{i}|F_{j}\right)$ is explained in Bouzembrak and Marvin 2016 [17] and Soon 2020 [21] and expressed as:

(1) $$P\left(F_{i}|F_{j}\right)=\frac{P\left(F_{j}|F_{i}\right)\times P\left(F_{i}\right)}{P\left(F_{j}\right)}$$

where $P\left(F_{i}\right)$ is the prior probability of event $F_{i}$, $P\left(F_{j}|F_{i}\right)$ is the probability of $F_{j}$ under the condition of known event $F_{i}$. $P\left(F_{j}\right)$ *P*(*F_j_*) is the probability of $F_{j}$. 

**Figure 2:**
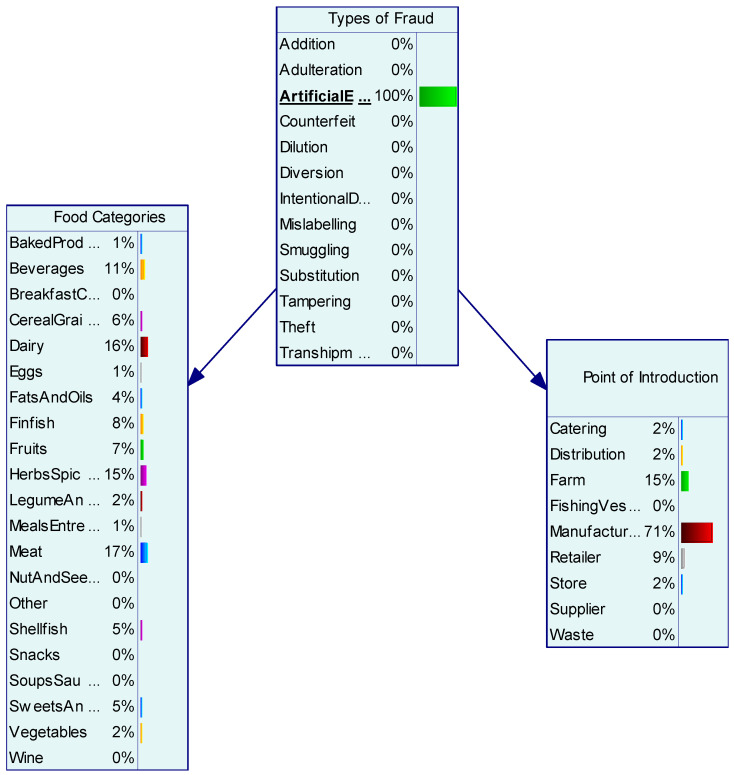
Artificial enhancement and prediction of specific food categories and point of adulteration.

Naïve-Bayes learning algorithm was used in the BN model. This is a simple BN structure that has the ‘Type of Food Fraud’ node as the parent node of all other nodes. As shown in Figure 2, the variables are connected by directed arcs. The arcs reflect the dependencies between each connected node. The nodes are differentiated as parent or child nodes. For example, there is a directed arc from ‘Type of Food Fraud’ to ‘Food Categories’, hence ‘Type of Food Fraud’ is the parent of ‘Food Categories’ and ‘Food Categories’ is the child node. Each node is associated with a probability distribution as a function of the states of the node’s parent variables. The variable $F_{i}$ with its parent—*pa*$\left(F_{i}\right)$ denotes the conditional probability distribution $P\left(F_{i}|pa\left(F_{i}\right)\right)$. The BN value is determined by combining the probability of all variables *P*(X) = *P*(*F*_1_….*F_n_*) by using the following formula [30]:

(2) $$P\left(Χ\right)=\;\prod_{i=1}^{n}\;P\left(F_{i}|pa\left(F_{i}\right)\right)$$

where Χ = combination of variables from $F_{1}$…$F_{n}$.

$Pa\left(F_{i}\right)$ = parent variable of $F_{i}$.

$P\left(F_{i}|pa\left(F_{i}\right)\right)\;$= probability of event $F_{i}$ under the condition of known parent $Pa\left(F_{i}\right)$.

**Table 1:** Types of fraud and explanatory variables.

Items	Description
Types of fraud *	Addition (Incorporation of cheaper ingredients to boost food/drink volume); adulteration ^ (modification of food or drink products—please see notes below); artificial enhancement (addition of unapproved chemical additives and/or addition of substances); counterfeit (exact copy of branded foods); dilution (reducing or thinning genuine drink products with cheaper ingredients); diversion (food products re-directed outside of intended markets); intentional distribution of unacceptable food (deliberate sale of unsafe or unacceptable food); mislabelling (misrepresentation of food/drink product), smuggling (illegal trade of food or drinks across borders), substitution (replacing genuine food products); tampering (for economic purposes); theft and transshipment (shipment and distribution of food/drinks to avoid tariffs)
Food categories	Baked products; beverages; breakfast cereals; cereal grains & pasta; dairy; eggs; fats & oils; finfish; fruits; herbs, spices & seasonings; legumes; meals, entrees & side dishes; meat & poultry; nut & seed products; other; shellfish; snacks; soups, sauces & gravies; sweets & confectionary; vegetables; wine
Year	1979–2018
Adulterants	Chemical (e.g., methanol, mineral oil, dye); ingredients (cheaper food ingredients); microbiological (e.g., Salmonella, E. coli; food subjected to temperature abuse); non-food (e.g., sewage water, animal feed, sand); other (e.g., mislabelling; smuggling; transshipment); physical (e.g., plastic crystals)
Reporting country	Worldwide
Point of adulteration	Catering; distribution (an intermediary between food producers and food operators such as retailers or restaurants and provides transportation of food); farm; fishing vessel; manufacturing; retailer (a place where consumers can buy food); store (warehouse); supplier; waste
Point of detection	Complaints; illnesses; inspections; investigation; other; raid; sampling; whistleblowing; not reported

* Based on [25,26,27,31]. ^ Adulteration was included when no indication of how the food or drink products were adulterated.

BN was developed using GeNie Modeler (BayesFusion, LLC, Pittsburgh, PA, USA) (http://www.bayesfusion.com/ (accessed on 10 August 2020)) with 715 (1979–2018) data points from the FAIR database.

### 2.3. Model Validation

The BN model was developed with data up to 2018 (715 cases) and validated with 80 cases from 2019. Two validations were carried out (i) type of food fraud; (ii) point of adulteration using the same 80 cases. The BN model computes the probabilities of food fraud type in relation to food or drinks categories and types of adulterants. All explanatory variables i.e., food categories, year, adulterants, reporting country, point of adulteration, and detection were used as input parameters in the BN model to predict food fraud type [18]. If the BN model predicts the fraud or point of adulteration correctly, a score of 1 was given, whilst 0 was given if the prediction was wrong.

## 3. Results

The BN model provides the distribution of probabilities for food fraud type as mislabelling (20.70%), followed by artificial enhancement (17.20%), substitution (16.36%), counterfeit (13.56%), and dilution (11.47%) (Figure S1).

Food and drink categories that were most commonly reported include beverages (21.40%), dairy (14.27%), meat (14.00%), fats & oils (9.37%), sweets and confectionery (7.55%), finfish (7.27%), and herbs, spices & seasonings (5.59%). Beverages were commonly produced as counterfeits (43.79%) or adulterated (19.60%) while dairy products were often diluted (35.29%) or artificially enhanced (19.60). Mislabelling (30%) and substitution (28%) were frequently reported in meat products. Fats and oils were mislabelled (28.35%) and substituted (26.86%).

Chemicals (21.68%) such as formaldehyde, mineral oil, methanol, and bleaching agent were used to adulterate food and drink products while ingredients (13.71%) that are cheaper or expired were used as forms of dilution and substitution. Microbiological hazards (13.01%) too were reported although—these were not intentionally added, however the incidences in such cases deliberately sale food products that were contaminated with pathogens.

Non-food products (4.20%) including sewage water, animal feed, and pet food were also used to bulk up or to substitute food and physical hazards (0.42%) including plastic crystals used to substitute sugar and googly eyes used to indicate the appearance of freshness in fish (by attaching fake googly eyes on the fish).

Other fraud notifications include intentional distribution of unacceptable food (6.57%), adulteration (5.45%), transhipment (3.08%), smuggling (2.52%), theft (1.96%), addition (0.56%), diversion (0.42%) and tampering (0.14%).

Manufacturing (63.92%) was identified as the main point of adulteration followed by the retailer (13.43%), distribution (9.93%), catering (5.73%), farm (4.62%), and others (2.37) i.e., storage, suppliers, fishing vessel and waste. During manufacturing, 19.04% (*n* = 457) of reports were categorized as an artificial enhancement. 18.60% were as a result of mislabelling and 17.29% were due to counterfeiting and substitution.

Food fraud activities during retailing were due to mislabelling (34.38%), substitution (16.67%), and intentional distribution of unacceptable food (16.67%). Transshipment (28.17%) and smuggling (22.53%) constituted the main reported fraud type during distribution. The point where the adulteration was detected was due to inspections (17.48%), investigations (13.85%), sampling (13.43%), and whistleblowing (11.19%). Most incidences do not report the point of detection (26.15%).

### 3.1. Validation of Food Fraud and Point of Adulteration Models

The BN food fraud prediction model was tested with 80 notifications from 2019 (Table S1). The correct food fraud type is shown in the fraud type column. The highest computed probability (%) was identified as the predicted fraud and this was noted as ‘1’ if detected correctly. The BN food fraud model predicted 63.8% of the fraud correctly. Table S2 predicts the point of adulteration based on 80 notifications from the same year. The model computes the probabilities of point of adulteration using food or drinks categories, types of adulterants, and food fraud type. The model predicted 71.3% of the point of adulteration correctly.

### 3.2. Application of BN Model

**Figure 3:**
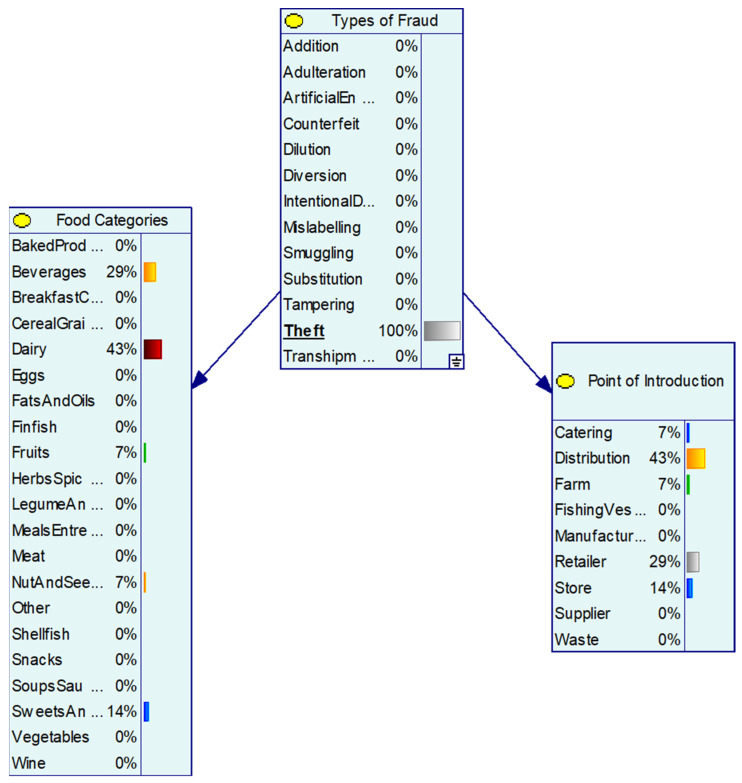
Theft and prediction of specific food categories and occurrence of theft in the supply chain.

To demonstrate the application of the BN model, the study used two case studies to exemplify the prediction of food categories affected by fraud and point of adulteration. Artificial enhancement predicted meat, dairy, and herbs & spices as the main food categories to be artificially enhanced using chemical additives and/or the addition of substances to improve the quality or attributes of the products. The point of adulteration (for artificial enhancement) occurred with the highest probability at manufacturing (71%) and farm sites (15%) (Figure 2). The theft was most commonly associated with dairy products (43%) and beverages (29%) and the BN model predicted the point of adulteration was highest during distribution (43%) followed by retailers (29%) and store (14%) (Figure 3). 

## 4. Discussion

The BN model revealed mislabelling as the most common form of food fraud. Mislabelling occurs when the food labeling information does not correspond to the actual content and production method of the food and drink product. Most of the mislabelling incidences identified in the BN model were linked to the country of origin, expiry date, nutritional information, and other credence attributes including organic, free-range, quality inspection, and religious dietary requirements. Misrepresentation of the provenance of meat products [32], olive oil [33], and seafood caught from the wild [34] have been reported. Country of origin was identified as an important factor when identifying possible fraud associated with food and drink products [2,18]. Similarly, Aboah and Lees 2020 [35] identified country of origin as the most important quality cue for beef and lamb products and organic and free-range labels as the most important for poultry. Fraudsters may take advantage of such attributes to boost their profits. This study corroborates with Li et al., 2020 [26], and Zhang and Xue, 2016 [27], who identified artificial enhancement as the major food fraud type and was due to usage of unapproved veterinary and human drug residues, or chemicals such as pesticides, food additives, and veterinary that were beyond the approved limits to enhance or prolonged the shelf-life of the food and drink products. Species replacement has been identified as a major problem in meat and poultry products [36,37], seafood [38,39], tubers [40], with 20% to more than 70% of the sampled products in the reported studies were identified as different species compared to the commercial name indicated on the label.

Beverages received the highest food fraud notifications as the drink category includes alcoholic beverages, fruit juices, tea, and coffee. According to Hong et al., 2017 [41], highly-priced beverages such as alcoholic drinks are likely targets (targeted) for food fraud. Fruit juices too are often subjected to adulteration as the method of adulteration is simple dilution with water or the addition of sugars and additives [42]. Adulteration of coffee and tea often involves the quality of raw materials (e.g., tea leaves and beans), substitution with other substances (e.g., chicory, coffee husks, low-grade tea leaves), artificially enhanced (e.g., color), or misrepresented as different geographical regions [43,44]. Notifications of adulteration of alcoholic beverages in this study were mostly due to the addition of industrial alcohol or the production of counterfeits. Alcoholic beverages were reported as one of the top processed commodities involved in food fraud [26]. Since the melamine scandal in 2008, dairy products have attracted considerable attention from public health authorities and various analytical detection methods [8] and prevention of fraud in the milk supply chain have been devised [9,45]. Dairy products are often diluted with extraneous water and then enhanced with nitrogen-rich adulterants to artificially boost the apparent protein content [8].

Chemicals and food-based ingredients were two of the most commonly reported adulterants used in this study. Such chemicals include formaldehyde, dyes, industrial alcohol, and veterinary residues. Similarly, in Zhang and Xue 2016 [27], more than 35% of the adulterants used were prohibited additives or additives that were added beyond the limits or inappropriately used. The various types of adulterants and highly diversified chemicals used in food are also emphasized in Everstine et al., (2018) [46]. The authors reported 1294 adulterants in the Decernis Food Fraud database of which 45% were categorized as potentially hazardous. 

Food processing has been identified as one of the most vulnerable points in the food supply chain as this is where alteration of food and drink products occurs making the food indistinguishable from its original form. This stage is particularly attractive to fraudsters as it enables raw materials to be modified by mixing with cheaper or low-quality ingredients or artificially enhanced with chemicals to boost the shelf-life, appearance, or nutritional content [47]. Within the beef supply chain, primary and secondary processing had been identified as the most vulnerable areas, of which counterfeiting (i.e., producing beef products in unapproved premised or without inspection) and species substitution took place [6]. Similarly, Everstine et al., 2013 [48] stated that processing techniques such as size reduction (e.g., grinding, chopping, milling) of herbs and spices represent the greatest risk of adulteration as the ground or crushed materials can hide the adulteration. This study identified two other main points of adulteration i.e., retailing and distribution. Most of the adulteration cases that were identified at the retailing point occurred at small and/or family-owned types of businesses. Food retailers were found to change the weight, expiry date, origin, and quality signs on the labels. Distribution was affected by transshipment and smuggling. Grabowski et al., 2013 and Soon and Manning 2018 [29,49] identified that products of animal origin especially exotic species were often smuggled into the EU as it forms part of the traditional diets of sub-cultures. Consumers’ demand for such food products encouraged smuggling via illegal distribution networks.

Inspections remain the main method in detecting fraudulent activities. Food safety inspections are carried out to ensure food businesses are complying with food laws and producing safe food. Inspections uncover fraudulent activities as authorized officers inspect the level of food hygiene and food standards, observe the premises and food safety management system, conduct sampling, or even follow up on complaints [50]. National food safety and sample inspection is an important method for detecting food fraud problems as demonstrated in China [26], Europe [17] including the Czech Republic [51], Finland [52], and Poland [53]. Food safety inspections that uncovered fraudulent activities could trigger follow-up investigations. Similarly, reports and/or complaints from customers and whistle-blowers could lead to inspections, raids, and investigations. Legislations are adopted by many countries to encourage and protect whistle-blowers especially against retribution [54]. The National Food Crime Unit (NCFU) established by the FSA, UK protects consumers from serious criminal activities and encourages food businesses, whistle-blowers, and consumers to report suspicions of food crime safely and confidentially [55].

The BN model is limited to FAIR data and predicted up to 64% of food fraud types and more than 70% of the point of adulteration correctly. These values were lower compared to previous studies derived from RASFF and EMA databases that predicted 80–91.5% of the fraud types correctly [17,18,21] The prediction could potentially be improved by combining with Decernis Food Fraud and HorizonScan databases to provide more accurate global food fraud prediction. Country of origin was not reported in all the incidents although this may be difficult due to the complexity and length of the food supply chain. It is recommended that the country of origin (of fraudulent activity) be included as this could help to improve the prediction of the origin of fraud. Food fraud notifications are also subjected to over- or under-reporting depending on countries and their ability to carry out food safety inspections and sampling. The BN model is based on historical data and hence will not be able to predict unknown and/or new types of food fraud and points of adulteration.

## 5. Conclusions

A BN model was developed based on the FAIR database which represents a snapshot of global food fraud incidences. Beverages, dairy, and meat products received the highest fraud notifications and were often processed as counterfeits, diluted, artificially enhanced, mislabelled, or substituted. Chemicals remained the favorite type of adulterants used to modify food and drink products. The model predicted 63.8% of the food fraud type and 71.3% of the point of adulteration correctly. BN models could be used to compute probability and provide practical implications for the food industry, food authorities, and researchers interested in food integrity. The BN model could be further applied to determine the food categories targeted by fraudsters. For example, how likely are meat, dairy, and herbs or spices artificially enhanced? Similarly, the BN model could be applied to predict the point of adulteration. Identifying the point of adulteration i.e., when food or drinks were intentionally modified or when fraudulent activities took place is the main and original contribution of this study. The ability to predict and identify the point of adulteration will enable targeted food fraud prevention plans and mitigating strategies. The prediction could be further enhanced by combining it with other global food fraud databases. However, food fraud databases must include (where possible) information on point of adulteration (e.g., at farm level, during primary or secondary processing, distribution or shipment, retailing, or catering). Prediction of food fraud type and point of adulteration could assist food safety inspections and regulatory control to target specific stages of the food supply chain and type of food to control and prevent food fraud from occurring.

## Data Availability

The data in the study are available within the article and in the Supplementary Material.

## Supplementary Materials

The following supporting information can be downloaded at https://www.mdpi.com/article/10.3390/foods11030328/s1, Figure S1: Bayesian Network (BN) model on food fraud; Table S1: BN model validation using 2019 data (*n* = 80); Table S2: BN model validation of point of adulteration (*n* = 80).
