# Supplementary material for: A Bayesian Approach to Predict Food Fraud Type and Point of Adulteration
Source: Foods. 2022 Jan 25;11(3):328. doi: 10.3390/foods11030328 (PMC8834205; doi:10.3390/foods11030328)
Supplement: Supplementary file 1 [file foods-11-00328-s001.zip › Table S1.pdf]

Table S1 BN model validation using 2019 data (n=80)

No.	Food and drink categories	Type of adulterants / Others	Fraud type	ADD	AD	AE	COU	DIL	DIV	INT	MIS	SMU	SUB	TAM	THE	TSP	Detected?
1	Dairy	Other	Theft	0	0.64	0	24.54	1.38	0	13.74	37.21	0	0.29	0	18.99	3.16	0
2	Sweets & confectionary	Other	Theft	0	0.39	0	14.77	0.74	0	8.27	26.13	3.81	0.13	0	7.62	38.11	0
3	Fats and oils	Other	Counterfeit	0	0.82	0	11.71	0.83	0	2.91	75.02	0	0.62	0	0	8.06	0
4	Herbs, spices and seasonings	Other	Mislabelling	0	0	0	34.59	0	0	17.21	34.97	11.89	1.32	0	0	0	1
5	Cereal grains and pasta	Other	Mislabelling	0	0	0	20.11	0	0	7.5	61.01	10.37	0.97	0	0	0	1
6	Meat	Other	Mislabelling	0	0	0	2.15	0	0	27.39	65.46	4.45	0.53	0	0	0	1
7	Finfish	Other	Mislabelling	0	0.34	0	3.28	0	0	2.45	69.83	16.97	0.31	0	0	6.78	1
8	Beverages	Other	Mislabelling	0	3.5	0	74.05	0.18	2.28	4.12	8.93	2.28	0.06	0	4.56	0	0
9	Cereal grains and pasta	Other	Intentional distribution of unacceptable food	0	0	0	20.11	0	0	7.5	61.01	10.37	0.97	0	0	0	0
10	Dairy	Other	Intentional distribution of unacceptable food	0	0.64	0	24.45	1.38	0	13.74	37.21	0	0.29	0	18.99	3.16	0
11	Fruits	Other	Intentional distribution of unacceptable food	0	0	0	20.81	0	0	15.53	42.08	0	0.09	0	10.73	10.73	0
12	Herbs, spices and seasonings	Non-food	Substitution	0	0	9.97	0	0	0	2.9	0	0	87.12	0	0	0	1
13	Snacks	Other	Intentional distribution of unacceptable food	0	0	0	0	0	0	100	0	0	0	0	0	0	1
14	Sweets & confectionary	Other	Intentional distribution of unacceptable food	0	0.39	0	14.77	0.74	0	8.27	26.13	3.81	0.13	0	7.62	38.11	0
15	Beverages	Other	Smuggling	0	3.5	0	74.05	0.18	2.28	4.12	8.93	2.28	0.06	0	4.56	0	0
16	Meat	Other	Intentional distribution of unacceptable food	0	0	0	2.15	0	0	27.39	65.46	4.45	0.53	0	0	0	0
17	Meals, entrees & side dishes	Other	Mislabelling	0	0	0	0	0	0	19.75	80.24	0	0	0	0	0	1

No.	Food and drink categories	Type of adulterants / Others	Fraud type	ADD	AD	AE	COU	DIL	DIV	INT	MIS	SMU	SUB	TAM	THE	TSP	Detected?
18	Meat	Other	Intentional distribution of unacceptable food	0	0	0	2.15	0	0	27.39	65.46	4.45	0.53	0	0	0	0
19	Sweets & confectionary	Chemical	Artificial enhancement	0	10.21	76.34	1.76	5.55	0	2.72	0	0	3.4	0	0	0	1
20	Beverages	Other	Smuggling	0	3.5	0	74.05	0.18	2.28	4.12	8.93	2.28	0.06	0	4.56	0	0
21	Beverages	Chemical	Adulteration	0	56.4	32.78	5.42	0.83	0	0.83	0	0	1.09	2.61	0	0	1
22	Sweets & confectionary	Other	Mislabelling	0	0.39	0	14.77	0.74	0	8.27	26.13	3.81	0.13	0	7.62	38.11	0
23	Egg	Other	Mislabelling	0	0	0	0	0	0	12.86	87.13	0	0	0	0	0	1
24	Beverages	Other	Counterfeit	0	3.5	0	74.05	0.18	2.28	4.12	8.93	2.28	0.06	0	4.56	0	1
25	Dairy	Other	Adulteration	0	0.64	0	24.54	1.38	0	13.74	37.21	0	0.29	0	18.99	3.16	0
26	Fats and oils	Chemical	Artificial enhancement	0	19	59.18	1.22	5.48	0	0.84	0	0	14.25	0	0	0	1
27	Herbs, spices and seasonings	Chemical	Artificial enhancement	0	0	94.16	0.54	0	0	0.74	0	0	4.54	0	0	0	1
28	Legume and legume products	Chemical	Artificial enhancement	0	26.38	65.73	1.13	0	0	2.34	0	0	4.39	0	0	0	1
29	Nut and seed products	Chemical	Artificial enhancement	0	0	0	0	0	0	0	0	0	100	0	0	0	0
30	Beverages	Chemical	Adulteration	0	56.4	32.78	5.42	0.83	0	0.83	0	0	1.09	2.61	0	0	1
31	Vegetables	Other	Mislabelling	0	0	0	7.26	0	0	10.85	36.74	15	0.12	0	0	30	1
32	Finfish	Other	Mislabelling	0	0.34	0	3.28	0	0	2.45	69.83	16.97	0.31	0	0	6.78	1
33	Beverages	Other	Mislabelling	0	3.5	0	74.05	0.18	2.28	4.12	8.93	2.28	0.06	0	4.56	0	0
34	Fruits	Other	Intentional distribution of unacceptable food	0	0	0	20.81	0	0	15.53	42.08	0	0.09	0	10.73	10.73	0
35	Meat	Other	Mislabelling	0	0	0	2.15	0	0	27.39	65.46	4.45	0.53	0	0	0	1
36	Fruits	Other	Mislabelling	0	0	0	20.81	0	0	15.53	42.08	0	0.09	0	10.73	10.73	1
37	Soups, sauces & gravies	Other	Mislabelling	0	4.01	0	37.98	0	0	0	38.4	0	0	0	0	19.59	1
38	Beverages	Other	Intentional distribution of unacceptable food	0	3.5	0	74.05	0.18	2.28	4.12	8.93	2.28	0.06	0	4.56	0	0
39	Herbs, spices and seasonings	Other	Counterfeit	0	0	0	34.59	0	0	17.21	34.97	11.89	1.32	0	0	0	0

No.	Food and drink categories	Type of adulterants / Others	Fraud type	ADD	AD	AE	COU	DIL	DIV	INT	MIS	SMU	SUB	TAM	THE	TSP	Detected?
40	Beverages	Other	Counterfeit	0	3.5	0	74.05	0.18	2.28	4.12	8.93	2.28	0.06	0	4.56	0	1
41	Finfish	Other	Mislabelling	0	0.34	0	3.28	0	0	2.45	69.83	16.97	0.31	0	0	6.78	1
42	Meals, entrees & side dishes	Other	Mislabelling	0	0	0	0	0	0	19.75	80.24	0	0	0	0	0	1
43	Meat	Other	Mislabelling	0	0	0	2.15	0	0	27.39	65.46	4.45	0.53	0	0	0	1
44	Meat	Other	Smuggling	0	0	0	2.15	0	0	27.39	65.46	4.45	0.53	0	0	0	0
45	Beverages	Chemical	Artificial enhancement	0	56.4	32.78	5.42	0.83	0	0.83	0	0	1.09	2.61	0	0	0
46	Fruits	Other	Mislabelling	0	0	0	20.81	0	0	15.53	42.08	0	0.09	0	10.73	10.73	1
47	Beverages	Other	Counterfeit	0	3.5	0	74.05	0.18	2.28	4.12	8.93	2.28	0.06	0	4.56	0	1
48	Dairy	Other	Mislabelling	0	0.64	0	24.54	1.38	0	13.74	37.21	0	0.29	0	18.99	3.16	1
49	Beverages	Non-food	Substitution	0	0	9.93	0	20.75	0	9.28	0	0	60.03	0	0	0	1
50	Herbs, spices and seasonings	Non-food	Substitution	0	0	9.97	0	0	0	2.9	0	0	87.12	0	0	0	1
51	Vegetables	Other	Mislabelling	0	0	0	7.26	0	0	10.85	36.74	15	0.12	0	0	30	1
52	Cereal grains and pasta	Chemical	Artificial enhancement	6.62	0	82.98	0.81	0	0	0.84	0	0	8.72	0	0	0	1
53	Dairy	Chemical	Artificial enhancement	0	6.68	83.23	1.15	4.08	0	1.78	0	0	3.06	0	0	0	1
54	Fats and oils	Ingredient	Dilution	0	2.14	2.43	0	93.1	0	0	2.3	0	0	0	0	0	1
55	Dairy	Other	Mislabelling	0	0.64	0	24.54	1.38	0	13.74	37.21	0	0.29	0	18.99	3.16	1
56	Meat	Other	Mislabelling	0	0	0	2.15	0	0	27.39	65.46	4.45	0.53	0	0	0	1
57	Meat	Other	Intentional distribution of unacceptable food	0	0	0	2.15	0	0	27.39	65.46	4.45	0.53	0	0	0	0
58	Fats and oils	Other	Adulteration	0	0.82	0	11.71	0.83	0	2.91	75.02	0	0.62	0	0	8.06	0
59	Cereal grains and pasta	Other	Counterfeit	0	0	0	20.11	0	0	7.5	61.01	10.37	0.97	0	0	0	0
60	Fats and oils	Ingredient	Artificial enhancement	0	2.14	2.43	0	93.1	0	0	2.3	0	0	0	0	0	0
61	Beverages	Chemical	Adulteration	0	56.4	32.78	5.42	0.83	0	0.83	0	0	1.09	2.61	0	0	1
62	Beverages	Other	Substitution	0	3.5	0	74.05	0.18	2.28	4.12	8.93	2.28	0.06	0	4.56	0	0
63	Dairy	Other	Mislabelling	0	0.64	0	24.54	1.38	0	13.74	37.21	0	0.29	0	18.99	3.16	1
64	Fats and oils	Other	Mislabelling	0	0.82	0	11.71	0.83	0	2.91	75.02	0	0.62	0	0	8.06	1
65	Cereal grains and pasta	Other	Smuggling	0	0	0	20.11	0	0	7.5	61.01	10.37	0.97	0	0	0	0

No.	Food and drink categories	Type of adulterants / Others	Fraud type	ADD	AD	AE	COU	DIL	DIV	INT	MIS	SMU	SUB	TAM	THE	TSP	Detected?
66	Vegetables	Other	Mislabelling	0	0	0	7.26	0	0	10.85	36.74	15	0.12	0	0	30	1
67	Meat	Chemical	Artificial enhancement	1.14	0	86.06	0.14	0	0	4.97	0	0	7.67	0	0	0	1
68	Dairy	Ingredient	Dilution	0	1.02	4.62	0	93.66	0	0	0.69	0	0	0	0	0	1
69	Dairy	Chemical	Artificial enhancement	0	6.68	83.23	1.15	4.08	0	1.78	0	0	3.06	0	0	0	1
70	Finfish	Ingredient	Artificial enhancement	0	12.65	57.32	0	0	0	0	30.01	0	0	0	0	0	1
71	Beverages	Chemical	Adulteration	0	56.4	32.78	5.42	0.83	0	0.83	0	0	1.09	2.61	0	0	1
72	Fats and oils	Ingredient	Dilution	0	2.14	2.43	0	93.1	0	0	2.3	0	0	0	0	0	1
73	Shellfish	Other	Mislabelling	0	0	0	0	0	0	5.32	64.95	22.09	0.25	0	0	7.36	1
74	Meat	Other	Mislabelling	0	0	0	2.15	0	0	27.39	65.46	4.45	0.53	0	0	0	1
75	Meat	Ingredient	Addition	24.46	0	55.69	0	0	0	0	19.83	0	0	0	0	0	0
76	Beverages	Other	Counterfeit	0	3.5	0	74.05	0.18	2.28	4.12	8.93	2.28	0.06	0	4.56	0	1
77	Fats and oils	Ingredient	Dilution	0	2.14	2.43	0	93.1	0	0	2.3	0	0	0	0	0	1
78	Vegetables	Other	Mislabelling	0	0	0	7.26	0	0	10.85	36.74	15	0.12	0	0	30	1
79	Finfish	Other	Smuggling	0	0.34	0	3.28	0	0	2.45	69.83	16.97	0.31	0	0	6.78	0
80	Vegetables	Other	Mislabelling	0	0	0	7.26	0	0	10.85	36.74	15	0.12	0	0	30	1

ADD=Addition; AD=Adulteration; AE=Artificial enhancement; COU=Counterfeit; DIL=Dilution; DIV=Diversion; INT=Intentional distribution of unacceptable food; MIS=Mislabelling; SMU=Smuggling; SUB=Substitution; TAM=Tampering; THE=Theft; TSP=Transhipment. Others include counterfeit, diversion, intentional distribution of unacceptable food, mislabelling, smuggling, theft, transhipment and unknown
