# Supplementary material for: A Bayesian Approach to Predict Food Fraud Type and Point of Adulteration
Source: Foods. 2022 Jan 25;11(3):328. doi: 10.3390/foods11030328 (PMC8834205; doi:10.3390/foods11030328)
Supplement: Supplementary file 1 [file foods-11-00328-s001.zip › Table S2.pdf]

Table S2 BN model validation of point of adulteration (n=80)

No.	Food and drink categories	Type of adulterants / Others	Fraud type	Point of Adulteration	CTR	DIS	FM	FV	MFC	RET	STO	SUP	WAS	Detected?
1	Dairy	Other	Theft	Distribution	7.14	42.86	7.14	0	0	28.57	14.29	0	0	1
2	Sweets & confectionary	Other	Theft	Distribution	7.14	42.86	7.14	0	0	28.57	14.29	0	0	1
3	Fats and oils	Other	Counterfeit	Manufacturing	4.12	5.15	0	0	81.44	7.22	1.03	1.03	0	1
4	Herbs, spices and seasonings	Other	Mislabelling	Manufacturing	8.78	6.76	4.05	0.67	57.43	22.29	0	0	0	1
5	Cereal grains and pasta	Other	Mislabelling	Manufacturing	8.78	6.76	4.05	0.67	57.43	22.29	0	0	0	1
6	Meat	Other	Mislabelling	Manufacturing	8.78	6.76	4.05	0.67	57.43	22.29	0	0	0	1
7	Finfish	Other	Mislabelling	Manufacturing	8.78	6.76	4.05	0.67	57.43	22.29	0	0	0	1
8	Beverages	Other	Mislabelling	Manufacturing	8.78	6.76	4.05	0.67	57.43	22.29	0	0	0	1
9	Cereal grains and pasta	Other	Intentional distribution of unacceptable food	Manufacturing	4.25	6.38	8.51	0	40.43	34.04	4.25	2.12	0	1
10	Dairy	Other	Intentional distribution of unacceptable food	Manufacturing	4.25	6.38	8.51	0	40.43	34.04	4.25	2.12	0	1
11	Fruits	Other	Intentional distribution of unacceptable food	Manufacturing	4.25	6.38	8.51	0	40.43	34.04	4.25	2.12	0	1
12	Herbs, spices and seasonings	Non-food	Substitution	Manufacturing	14.53	0.85	1.71	0	67.52	13.66	0.85	0.85	0	1
13	Snacks	Other	Intentional distribution of unacceptable food	Manufacturing	4.25	6.38	8.51	0	40.42	34.04	4.25	2.12	0	1
14	Sweets & confectionary	Other	Intentional distribution of unacceptable food	Manufacturing	4.25	6.38	8.51	0	40.42	34.04	4.25	2.12	0	1
15	Beverages	Other	Smuggling	Distribution	0	88.89	0	5.56	5.56	0	0	0	0	1
16	Meat	Other	Intentional distribution of unacceptable food	Manufacturing	4.26	6.38	8.51	0	40.42	34.04	4.26	2.12	0	1
17	Meals, entrees & side dishes	Other	Mislabelling	Catering	8.78	6.76	4.05	0.68	57.43	22.29	0	0	0	0

No.	Food and drink categories	Type of adulterants / Others	Fraud type	Point of Adulteration	CTR	DIS	FM	FV	MFC	RET	STO	SUP	WAS	Detected?
18	Meat	Other	Intentional distribution of unacceptable food	Manufacturing	4.26	6.38	8.51	0	40.42	34.04	4.26	2.12	0	1
19	Sweets & confectionary	Chemical	Artificial enhancement	Manufacturing	1.63	1.63	15.44	0	70.73	8.94	1.63	0	0	1
20	Beverages	Other	Smuggling	Distribution	0	88.89	0	5.56	5.56	0	0	0	0	1
21	Beverages	chemical	adulteration	Manufacturing	0	2.56	0	0	92.31	2.56	0	2.56	0	1
22	Sweets & confectionary	Other	Mislabelling	Manufacturing	8.78	6.76	4.05	0.68	57.43	22.29	0	0	0	1
23	egg	Other	Mislabelling	Retailer	8.78	6.76	4.05	0.68	57.43	22.29	0	0	0	0
24	Beverages	Other	Counterfeit	Manufacturing	4.12	5.15	0	0	81.44	7.22	1.03	1.03	0	1
25	Dairy	Other	Adulteration	Manufacturing	0	2.56	0	0	92.31	2.56	0	2.56	0	1
26	Fats and oils	Chemical	Artificial enhancement	Manufacturing	1.63	1.63	15.44	0	70.73	8.94	1.63	0	0	1
27	Herbs, spices and seasonings	Chemical	Artificial enhancement	Manufacturing	1.63	1.63	15.44	0	70.73	8.94	1.63	0	0	1
28	Legume and legume products	Chemical	Artificial enhancement	Manufacturing	1.63	1.63	15.44	0	70.73	8.94	1.63	0	0	1
29	Nut and seed products	Chemical	Artificial enhancement	Manufacturing	1.63	1.63	15.44	0	70.73	8.94	1.63	0	0	1
30	Beverages	Chemical	Adulteration	Manufacturing	0	2.56	0	0	92.31	2.56	0	2.56	0	1
31	Vegetables	Other	Mislabelling	Retailer	8.78	6.76	4.05	0.68	57.43	22.29	0	0	0	0
32	Finfish	Other	Mislabelling	Manufacturing	8.78	6.76	4.05	0.68	57.43	22.29	0	0	0	1
33	Beverages	Other	Mislabelling	Manufacturing	8.78	6.76	4.05	0.68	57.43	22.29	0	0	0	1
34	Fruits	Other	Intentional distribution of unacceptable food	Retailer	4.26	6.38	8.51	0	40.42	34.04	4.26	2.12	0	0
35	Meat	Other	Mislabelling	Retailer	8.78	6.76	4.05	0.68	57.43	22.29	0	0	0	0
36	Fruits	Other	Mislabelling	Farm	8.78	6.76	4.05	0.68	57.43	22.29	0	0	0	0
37	Soups, sauces & gravies	Other	Mislabelling	Farm	8.78	6.76	4.05	0.68	57.43	22.29	0	0	0	0
38	Beverages	Other	Intentional distribution of unacceptable food	Manufacturing	4.26	6.38	8.51	0	40.42	34.04	4.26	2.12	0	1
39	Herbs, spices and seasonings	Other	Counterfeit	Manufacturing	4.12	5.15	0	0	81.44	7.22	1.03	1.03	0	1

No.	Food and drink categories	Type of adulterants / Others	Fraud type	Point of Adulteration	CTR	DIS	FM	FV	MFC	RET	STO	SUP	WAS	Detected?
40	Beverages	Other	Counterfeit	Manufacturing	4.12	5.15	0	0	81.44	7.22	1.03	1.03	0	1
41	Finfish	Other	Mislabelling	Retail	8.78	6.76	4.05	0.68	57.43	22.29	0	0	0	0
42	Meals, entrees & side dishes	Other	Mislabelling	Retail	8.78	6.76	4.05	0.68	57.43	22.29	0	0	0	0
43	Meat	Other	Mislabelling	Retailer	8.78	6.76	4.05	0.68	57.43	22.29	0	0	0	0
44	Meat	Other	Smuggling	Distribution	0	88.89	0	5.56	5.56	0	0	0	0	1
45	Beverages	Chemical	Artificial enhancement	Manufacturing	1.63	1.63	15.44	0	70.73	8.94	1.63	0	0	1
46	Fruits	Other	Mislabelling	Supplier	8.78	6.76	4.05	0.68	57.43	22.29	0	0	0	0
47	Beverages	Other	Counterfeit	Manufacturing	4.12	5.15	0	0	81.44	7.22	1.03	1.03	0	1
48	Dairy	Other	Mislabelling	Manufacturing	8.78	6.76	4.05	0.68	57.43	22.29	0	0	0	1
49	Beverages	Non-food	Substitution	Manufacturing	14.53	0.85	1.71	0	67.52	13.68	0.85	0.85	0	1
50	Herbs, spices and seasonings	Non-food	Substitution	Distribution	12.94	1.09	3.28	0	67.06	13.79	1.03	0.81	0	0
51	Vegetables	Other	Mislabelling	Farm	8.78	6.76	4.05	0.68	57.43	22.29	0	0	0	0
52	Cereal grains and pasta	Chemical	Artificial enhancement	Manufacturing	1.63	1.63	15.44	0	70.73	8.94	1.63	0	0	1
53	Dairy	Chemical	Artificial enhancement	Manufacturing	1.63	1.63	15.44	0	70.73	8.94	1.63	0	0	1
54	Fats and oils	Ingredient	Dilution	Manufacturing	2.44	8.54	1.22	0	82.93	4.88	0	0	0	1
55	Dairy	Other	Mislabelling	Retailer	8.78	6.76	4.05	0.68	57.43	22.29	0	0	0	0
56	Meat	Other	Mislabelling	Retailer	8.78	6.76	4.05	0.68	57.43	22.29	0	0	0	0
57	Meat	Other	Intentional distribution of unacceptable food	Retailer	4.26	6.38	8.51	0	40.42	34.04	4.26	2.12	0	0
58	Fats and oils	Other	Adulteration	Manufacturing	0	2.56	0	0	92.31	2.56	0	2.56	0	1
59	cereal grains and pasta	Other	Counterfeit	Manufacturing	4.12	5.15	0	0	81.44	7.22	1.03	1.03	0	1
60	Fats and oils	Ingredient	Artificial enhancement	Manufacturing	1.63	1.63	15.44	0	70.73	8.94	1.63	0	0	1
61	Beverages	Chemical	Adulteration	Manufacturing	0	2.56	0	0	92.31	2.56	0	2.56	0	1
62	Beverages	Other	Substitution	Manufacturing	14.53	0.85	1.71	0	67.52	13.68	0.85	0.85	0	1
63	Dairy	Other	Mislabelling	Distribution	8.78	6.76	4.05	0.68	57.43	22.29	0	0	0	0
64	Fats and oils	Other	Mislabelling	Distribution	8.78	6.76	4.05	0.68	57.43	22.29	0	0	0	0
65	Cereal grains and pasta	Other	Smuggling	Distribution	0	88.89	0	5.56	5.56	0	0	0	0	1

No.	Food and drink categories	Type of adulterants / Others	Fraud type	Point of Adulteration	CTR	DIS	FM	FV	MFC	RET	STO	SUP	WAS	Detected?
66	Vegetables	Other	Mislabelling	Manufacturing	8.78	6.76	4.05	0.68	57.43	22.29	0	0	0	1
67	Meat	Chemical	Artificial enhancement	Farm	1.63	1.63	15.44	0	70.73	8.94	1.63	0	0	0
68	Dairy	Ingredient	Dilution	Retailer	2.44	8.54	1.22	0	82.93	4.88	0	0	0	0
69	Dairy	Chemical	Artificial enhancement	Manufacturing	1.63	1.63	15.44	0	70.73	8.94	1.63	0	0	1
70	Finfish	Ingredient	Artificial enhancement	Manufacturing	1.63	1.63	15.44	0	70.73	8.94	1.63	0	0	1
71	Beverages	Chemical	Adulteration	Manufacturing	0	2.56	0	0	92.31	2.56	0	2.56	0	1
72	Fats and oils	Ingredient	Dilution	Manufacturing	2.44	8.54	1.22	0	82.93	4.88	0	0	0	1
73	Shellfish	Other	Mislabelling	Manufacturing	8.78	6.76	4.05	0.68	57.43	22.29	0	0	0	1
74	Meat	Other	Mislabelling	Retailer	8.78	6.76	4.05	0.68	57.43	22.29	0	0	0	0
75	Meat	Ingredient	Addition	Manufacturing	0	0	0	0	75	25	0	0	0	1
76	Beverages	Other	Counterfeit	Manufacturing	4.12	5.15	0	0	81.44	7.22	1.03	1.03	0	1
77	Fats and oils	Ingredient	Dilution	Manufacturing	2.44	8.54	1.22	0	82.93	4.88	0	0	0	1
78	Vegetables	Other	Mislabelling	Retailer	8.78	6.76	4.05	0.68	57.43	22.29	0	0	0	0
79	Finfish	Other	Smuggling	Distribution	0	88.89	0	5.56	5.56	0	0	0	0	1
80	Vegetables	Other	Mislabelling	Distribution	8.78	6.76	4.05	0.68	57.43	22.29	0	0	0	0

CTR=Catering; DIS=Distribution; FM=Farm; FV=Fishing vessel; MFC=Manufacturing; RET=Retailer; STO=Store; SUP=Supplier; WAS=Waste. Others include counterfeit, diversion, intentional distribution of unacceptable food, mislabelling, smuggling, theft, transhipment and unknown
