# Supplementary figures and images for: A Bayesian Approach to Predict Food Fraud Type and Point of Adulteration
Source: Foods. 2022 Jan 25;11(3):328. doi: 10.3390/foods11030328 (PMC8834205; doi:10.3390/foods11030328)

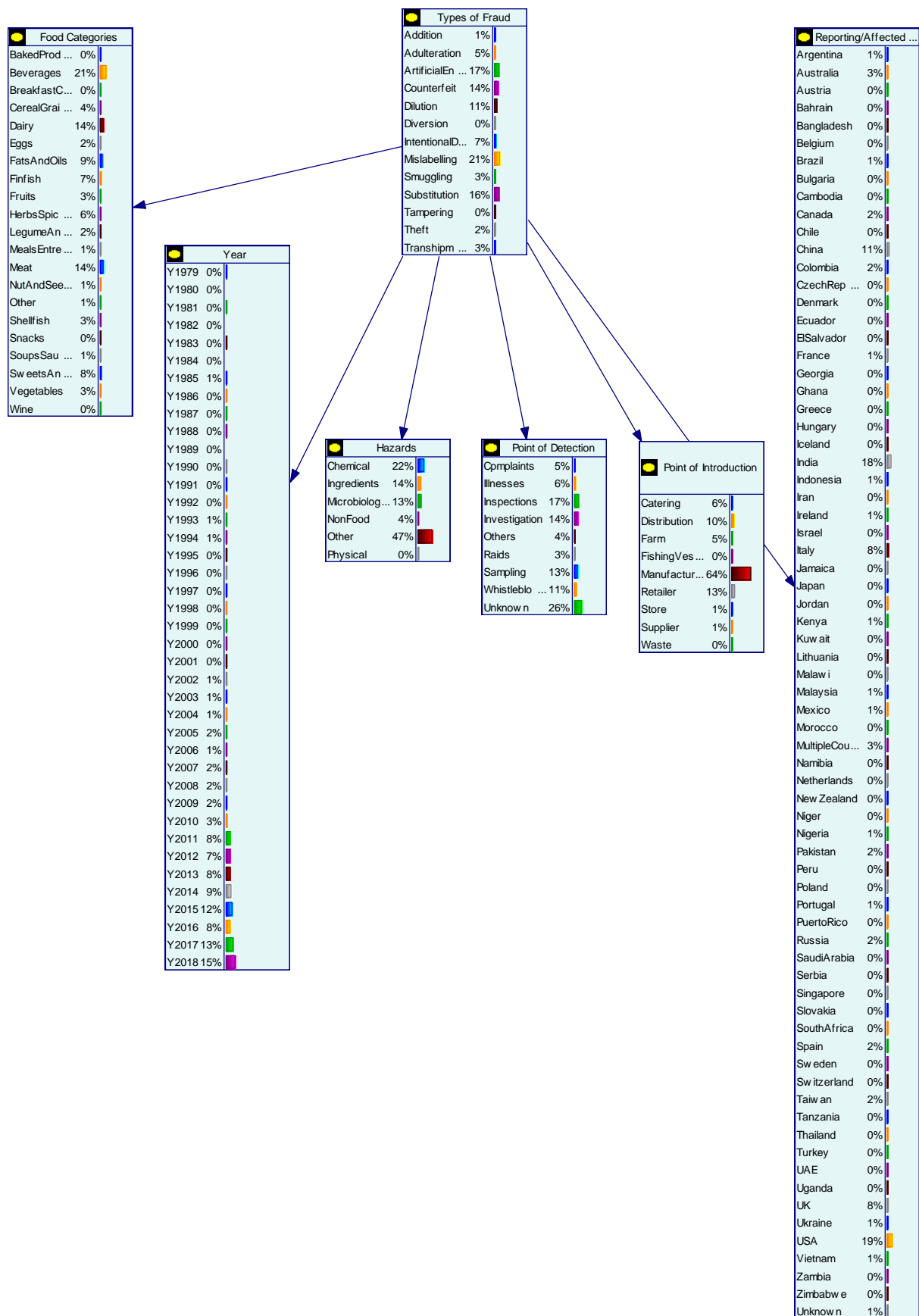

Supplementary Material Figure 1. Bayesian Network (BN) model on food fraud

Supplement: Supplementary file 1 [file foods-11-00328-s001.zip › Figure S1.pdf]
